# Olfaction-based learned preference assessment without the use of motivational fear or motivational weight loss

**DOI:** 10.3389/fnbeh.2025.1521751

**Published:** 2025-02-12

**Authors:** Sara E. Moss, Ekaterina S. McCurdy, Natalya N. Thomas, Danielle Gulick, Angela M. Poff, Dominic P. D'Agostino

**Affiliations:** ^1^Department of Molecular Pharmacology and Physiology, University of South Florida, Tampa, FL, United States; ^2^Molecular Medicine, University of South Florida, Tampa, FL, United States

**Keywords:** olfactory, metabolic therapy, learning, taste, LPS

## Abstract

**Introduction:**

Reliable assessments of learning ability in preclinical models are essential for studying neurodegenerative, developmental, and inflammatory disorders. However, many inbred strains of mice present background pathologies that interfere with traditional learning tests. The C57BL/6 J mouse, a widely used laboratory strain, sporadically develops auditory and visual impairments that complicate interpretation. In this study, we establish an olfaction-based learned preference protocol designed to evaluate learning ability independent of fear responses, motivational weight loss, or visual cues in C57BL/6 J mice.

**Methods and results:**

Leveraging the species’ natural preference for sweet flavors, we tested different sweeteners and confirmed their passive preference for sucrose was more robust than for saccharin or sucralose. We then trained mice to associate either lemon or rose scents with a sucrose paste reward, and tested whether they demonstrated a learned preference for the sucrose-associated scent over the neutral scent. Mice developed an appetitive olfactory preference for sucrose as a reward, in the absence of motivational weight loss, as measured by time spent exploring a three-chamber association box with access to both scents. We assessed whether this protocol discriminated learning deficit induced by lipopolysaccharide (LPS) administration.

**Conclusion:**

We conclude that this protocol is a viable tool for assessing learning abilities in preclinical models with auditory or visual deficits, motor impairments, or an inability to tolerate motivational weight loss.

## 1 Introduction

Preclinical mouse models are crucial tools for evaluating treatment effectiveness in disease studies. Learning ability assessments are often employed as indicators to monitor disease progression and measure the potential adverse effects of treatments (Othman et al., 2022; Vorhees and Williams, 2006; Lueptow, 2017; Jones et al., 2005; Shiotsuki et al., 2010). However, the specific genetic characteristics of some model systems can introduce confounding factors when assessing learning in mice, requiring careful consideration. Three significant factors impacting learning assessments are motor function, sensory (auditory and visual) capabilities, and motivation levels (Brown and Wong, 2007; Othman et al., 2022).

Motor function-based learning assessments, such as Morris Water Maze (MWM), assess the subject’s ability to learn a physical task (Vorhees and Williams, 2006). Measurements of how quickly the subjects learn to perform the task are used to determine learning ability. Differences in motor function, such as changes in body weight, muscle composition, and gait, confound the results of these assessments and can provide inaccurate measures of learning (Vorhees and Williams, 2006; Shiotsuki et al., 2010). Motor function-based learning assessments such as MWM and Rotarod tests are inherently stressful on the subjects, leading to potential variability in subject response (Sankowski et al., 2019; Hung and Hsueh, 2021). In cases where motor deficits are central to a disease phenotype or treatment effect, other learning assessments need to be used.

Vision-based learning assessments, such as Novel Object Recognition, assess the subject’s ability to recognize and respond to visual cues (Lueptow, 2017). Changes in visual capability and visuospatial awareness can affect performance in these tasks and can undermine the utility of this test in model systems that demonstrate such deficits. For example, C57BL/6 J mice, one of the most commonly used strains of laboratory mice, can develop sporadic and age related eye abnormalities even though they are considered to have “normal” vision overall and are used for visual based learning (Koch and Gowen, 1939; Ferdous et al., 2021; Ikeda et al., 1999; Brown and Wong, 2007; Berger et al., 2014). C57BL/6 J mice have also been shown to perform worse in visual tasks at 6 months of age than other strains (Wong and Brown, 2007). Other strains of mice, such as BALB/cJ and A/J, have established strain-wide disruptions in visual acuity that reduce their ability to learn via visual based assays (Brown and Wong, 2007; Wong and Brown, 2006). Visual capability can also be affected by various disorders, including inflammatory responses (Noailles et al., 2018). In cases where visual capabilities are affected by a disease phenotype or treatment effect, other learning assessments need to be used.

While many olfaction-based assessment protocols rely on visual cues or motor function, they can also offer an alternative option that can be used to avoid confounds with deficits in motor function or visual capability (Aqrabawi and Kim, 2018; Jones et al., 2005; Schellinck et al., 2001). However, these protocols commonly use fear response or motivational weight loss to increase participation levels during training so that learning capabilities can be assessed (Schellinck et al., 2001; Jones et al., 2005; Hakim et al., 2019). Fear conditioning is inherently stressful for the subject, which can create variability in subject responses. Fear conditioning is also often dependent on auditory and visual cues, leading to increased variability in subject responses (Xiao et al., 2018; Kuboyama et al., 2020). For example, if a treatment is anxiolytic, then it will reduce fear learning independent of learning capability. Motivational weight loss is an effective way to increase subject participation in food reward-based training, as it involves restricting food intake to induce weight loss in subjects (Schellinck et al., 2001). This restriction increases exploratory behavior which increases participation in tasks that are reward-based. However, as metabolism-based mechanisms of action are becoming more widely appreciated in health and disease, it is clear that caloric restriction and weight loss can present a confounding factor for many studies, especially those testing metabolic-targeted therapies (Riddle et al., 2013). Strains such as SPRET/EiJ mice are intolerant to motivational weight loss, and were excluded in an arm of a study comparing strain differences in learning capabilities due to this (Brown and Wong, 2007). This is likely due to variations in mouse strain body composition (Reed et al., 2007). In addition, anhedonia can suppress reward-seeking independent of learning. This makes using motivational weight loss inappropriate in studies for which changes in metabolism, changes in mood, or weight loss may be a central part of the disease process.

While learning assessment protocols exist that can overcome some of these confounding factors, there is a need for additional protocols that are independent of vision, audition, and aversive or caloric conditioning. Thus, we have designed and assessed an olfaction-based learning assessment that does not rely on motivational fear or weight loss. After demonstrating that C57BL/6 J mice can learn this task, we use lipopolysaccharide (LPS) administration (a model for sepsis that causes an inflammation-induced learning deficit in low doses; Zhao et al., 2019; Frühauf et al., 2015; Jung et al., 2023) as proof of concept to assess learning changes in this novel protocol. Effects of LPS treatment include changes in motor function and muscle composition, dramatic weight loss, and increases retinal inflammation (Skrzypczak-Wiercioch and Sałat, 2022; Zhang et al., 2022). Thus, we demonstrate a novel method for assessing olfaction-based learned preference in C57BL/6 J mice without the use of motivational fear or weight loss following LPS treatment.

## 2 Materials and equipment

### 2.1 Animal husbandry

**Figure 1:**
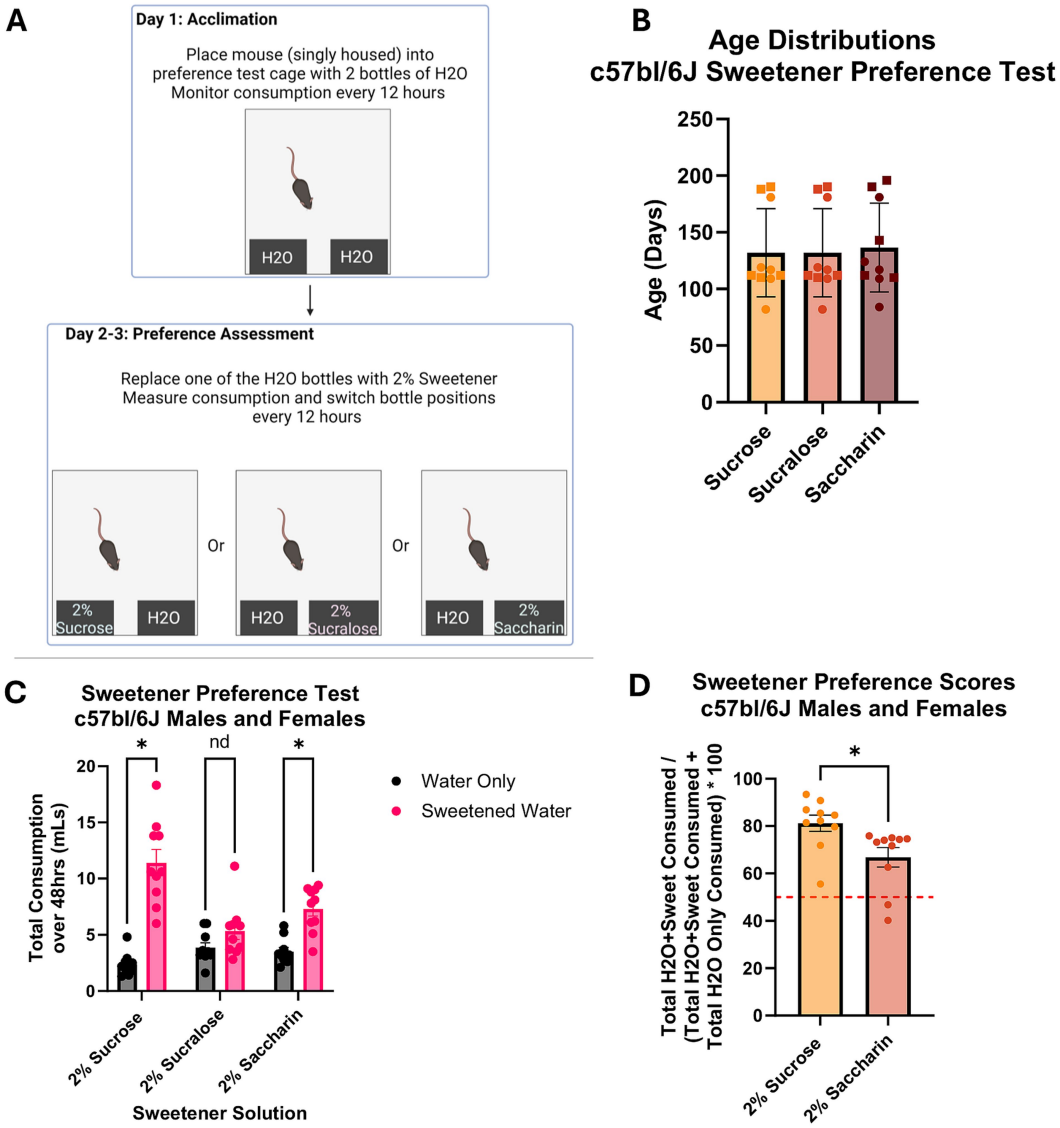
Sweetener preference assessment in C57BL/6 J Mice. **(A)** Diagram of sweetener preference assessment protocol *n* = 5f (squares) and 5 m (circles) for all groups. **(B)** Age distributions of mice assessed. Results of 2-way ANOVA show effect of sex or group assignment on mice age. **(C)** Total consumption of water and sweetened solutions. Results of 2-way ANOVA analyses show mice consumed more sucrose and saccharin solutions than water only solutions (*p* < 0.05). There was no difference in consumption between water and sucralose sweetened solution. **(D)** Preference scores of 2% sucrose and 2% saccharin solutions based on the amount consumed by the mice. Results of one-tailed t-test analyses show that mice exhibited a higher preference score for sucrose than saccharin (*p* < 0.05). A score of 50% is indicated by the red dotted line.

**Figure 2:**
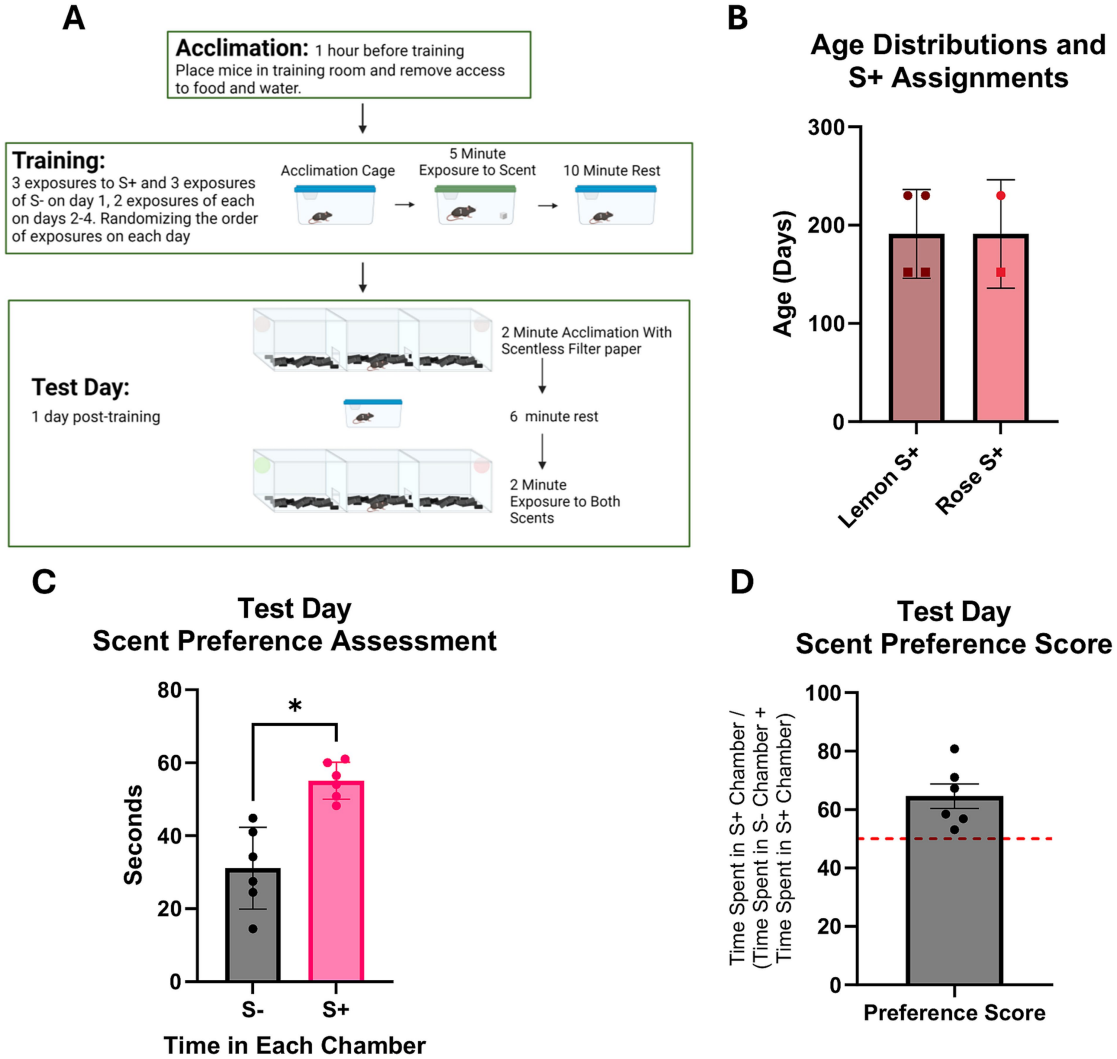
Olfaction based learned preference assessment in C57BL/6 J mice. *N* = 3f, 3 m. **(A)** Diagram of olfaction based learned preference assessment protocol. **(B)** Age distributions of mice assessed *n* = 3f (squares) and 3 m (circles). One male and one female were assigned rose as their S+ scent. **(C)** Total time spent in each scent containing chamber. S- indicates the scent not associated with the sucrose paste, and S+ indicates the scent associated with the sucrose paste. Results of paired t-test analyses show mice spent more time in the chamber containing S+ scent than in the chamber containing S- scent (*p* < 0.05). **(D)** Preference score of S+ chamber over the S- chamber based on the amount of time spent in each chamber. A score of 50% is indicated by the red dotted line.

**Figure 3:**
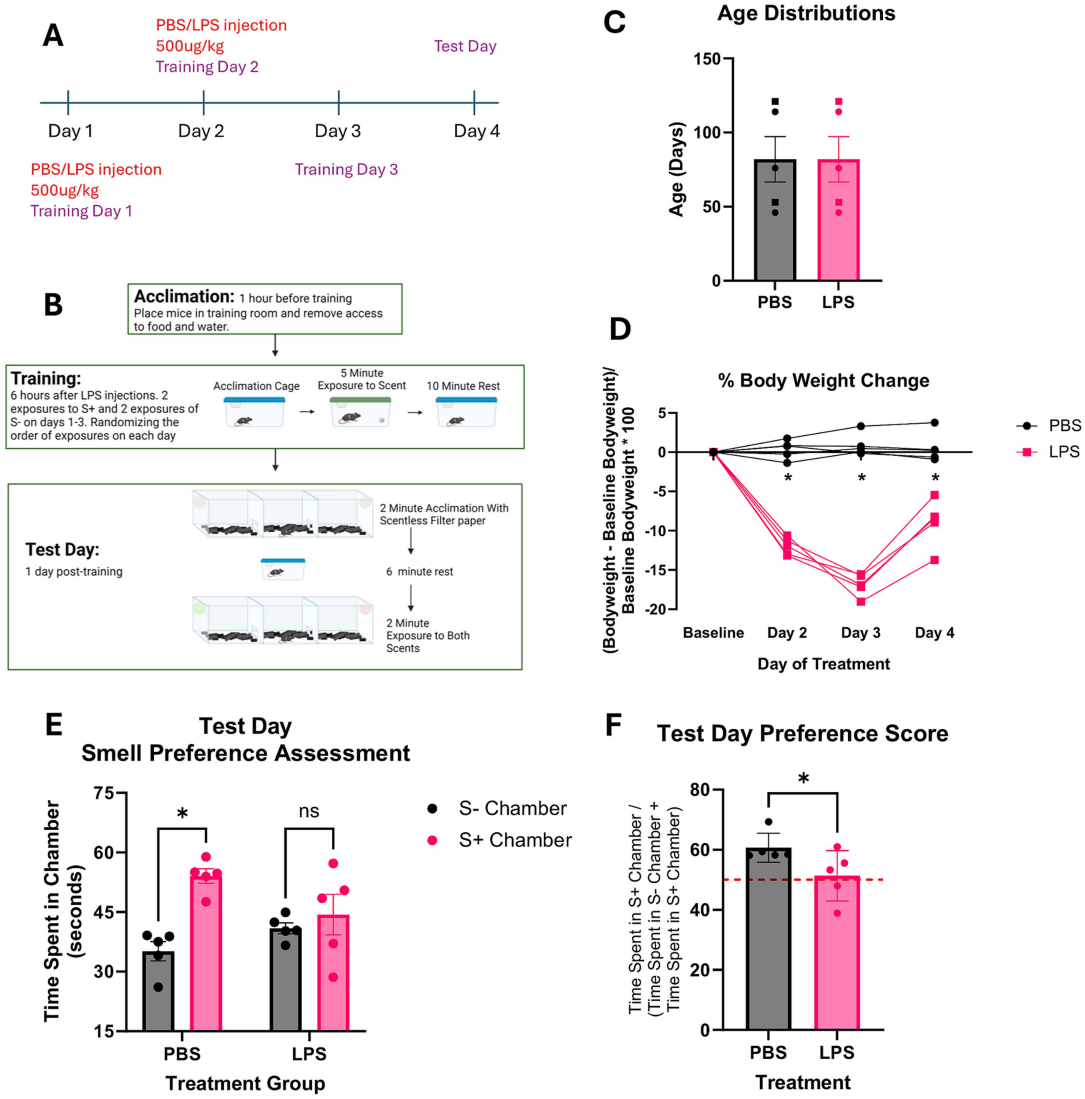
LPS induced learning deficit assessment in C57BL/6 J mice. **(A,B)** Visual timeline of LPS injections and olfaction based learned preference assessment protocol. **(C)** Age distributions and group assignments of mice assessed *n* = 2f (squares) and 3 m (circles) for each group. **(D)** %Body weight change from baseline following LPS/PBS treatments. **(E)** Total time spent in each scent containing chamber. S- indicates the scent not associated with the sucrose paste, and S+ indicates the scent associated with the sucrose paste. Results of repeated measures 2-way ANOVA analyses show PBS treated mice spent more time in the chamber containing S+ scent than in the chamber containing S- scent (*p* < 0.05) and LPS mice had no difference in time spent in S+ compared to S-. **(F)** Preference score of S+ chamber over the S- chamber based on the amount of time spent in each chamber. Results of paired t-test analyses show that PBS treated mice exhibited a higher preference score for the S+ chamber than the LPS treated mice (*p* < 0.05). A score of 50% is indicated by the red dotted line.

Male and female C57BL/6 J mice (between 7 and 33 weeks of age as indicated in Figures 1–3) were bred in-house in the University of South Florida Morsani College of Medicine Vivarium with a standard 12 h light/dark cycle. All animals were provided ad-libitum access to food and water except during specified training sessions. Sex and age distributions are shown in Figures 1–3. Ethics Statement: All procedures were completed within strict adherence to the NIH Guide for the Care and Use of Laboratory Animals and were approved by the University of South Florida Institutional Animal Care and Use Committee (IACUC; protocol number IS00009972).

### 2.2 Sweetener preference test

2% sweetener solutions were made by adding 2 g of sweetener [Sucrose (Fisher Chemical), Sucralose (ThermoScientific), or Saccharin (ThermoScientific)] to 98 mL of water. IVC cages with two sipper bottles per cage were used to present solutions to the mice using 50 mL of water or sweetener solutions per bottle.

### 2.3 Olfactory learned preference test

Sucrose Paste (85% Sucrose: 15% water by volume) was added to the bottom of the training cages using a ¼ teaspoon measuring spoon. Fresh scents were made every day using the following formulations:

1. Lemon: 15% linalool (Thermo Fisher Scientific) in propylene glycol (Fisher Chemical)
2. Rose: 15% phenyl acetate (Acros Organics) in propylene glycol

Scent Papers were prepared by cutting 46 mm diameter cellulose 8um filter papers in half, and adding 50uL of designated scent.

Two clean IVC housing cages per mouse per training day to use for training. Each mouse had one rose and one lemon training cage per training day, with fresh sucrose paste and fresh scented filter papers for each training trial. Each cage had enough bedding to cover the bottom of the cage. S+ cages had ¼ tsp. of sucrose paste added to the bottom of the back of the cage, underneath the scented filter paper. The scented filter paper was placed underneath the IVC cage’s air filter where it could be held in place out of the mouse’s reach, while still being able to diffuse the scent into the cage.

A clear acrylic 3 chambered association box (69 × 20 × 20 cm), with 6×5.5 cm openings in the internal dividing walls to allow access between the 3 chambers was used for test day, along with a video recording device.

### 2.4 LPS injections

Aliquots of LPS (Sigma Aldrich) dissolved in 1x PBS were stored at −80 and thawed to room temperature before injections. LPS was administered intraperitoneally using 3 mL insulin syringes at 500ug/Kg dosing based on the weight of the mouse measured using a scale on the day of injection.

## 3 Methods

### 3.1 Sweetener preference assessment

#### 3.1.1 Day 1: Acclimation

Mice were placed in isolated IVC cages with ad libitum access to food and two sipper bottles filled with water only. At hour 12, intake was measured and bottles were filled with fresh water. Bottle orientation was switched at hour 12.

#### 3.1.2 Day 2 + 3: Sweetener preference assessment

At hour 24, intake was measured and then mice were given 1 fresh water only bottle and 1 fresh sweetener solution bottle. At hours 36, 48, 60, and 72, intake was measured and bottles were refilled. Bottle orientation was switched at every refill to avoid orientation preference. Total intake of water only and sweetener solution was calculated and used for preference assessment.

### 3.2 Olfaction-based learned preference protocol

#### 3.2.1 Training days

Mice were placed in training room 1 h before training for acclimation. During acclimation, food and water were removed and were not replaced until the end of that training period. Training began 2 h before the end of the light cycle. One training trial consisted of placing the mice in a training cage for 5 min, then returning the mice to their acclimation cages for 10 min of rest before the next training trial. The order of scents during training days was randomized. On day 1, mice received three trials with lemon and three trials with rose scents. During the third training trial on day 1, mice began showing signs of overtraining which included resting, remaining stationary, or engaging in excessive grooming without exploration. On days 2–4, mice received two trials with lemon and two trials with rose scents. On day 5, smell preference was assessed.

#### 3.2.2 Smell preference assessment

Mice were placed in an acclimation room 1 h before training for acclimation. During acclimation, food and water were removed and were not replaced until the end of that testing period. Testing began 2 h before the end of the light cycle.

Using a clear acrylic 3 chambered association box (69 × 20 × 20 cm), with 6×5.5 cm openings in the internal dividing walls to allow access between the 3 chambers, un-scented filter papers were taped to the top of the 2 outermost chambers out of the mouse’s reach. Enough bedding was added to cover the bottom of the chamber. Mice were given 2 min of acclimation in the unscented chamber, then placed in their acclimation cages for 5 min inside the testing room. The filter papers were taken outside of the testing room to a smell-prep station away from the mice, without disturbing the orientation of the association chamber. 50uL of rose and lemon scents were added to the designated filter papers, and then placed back in the association chamber when the mouse’s resting period was completed. The mice were then placed in the center of the association chamber and a video recording was taken of their 2 min preference test. Between mice, the association chamber was cleaned with 40% ethanol to ensure no remaining scent lingered for the next mouse, and the chamber was rotated 180 degrees to limit any side preference the mice may have had. Videos were then scored by a person blinded to treatment conditions to determine the time spent in each smell chamber during the 2 min test.

### 3.3 Assessment of LPS-induced learning deficit

#### 3.3.1 LPS injections

Mice were weighed 30 min before LPS (Sigma Aldrich) injection at a dose of 500ug/Kg. LPS was suspended in PBS and administered via intraperitoneal (IP) injection. PBS was administered via IP injection at 500ug/Kg in control mice. Mice were continuously monitored and weighed throughout the study to ensure their safety. Injections were administered 6 h before training on days 1 and 2.

#### 3.3.2 Assessing olfaction-based learning deficit

##### 3.3.2.1 Training days

Mice were placed in the training room 1 h before training for acclimation. During acclimation, food and water were removed and were not replaced until the end of that training period. Training began 2 h before their change in light cycle. One training trial consisted of placing the mice in a training cage for 5 min, then returning the mice to their acclimation cages for 10 min of rest before the next training trial. The order of scents during training days was randomized. On days 1–3, mice received two trials with lemon and 2 trials with rose scents.

##### 3.3.2.2 Smell preference assessment

Mice were placed in an acclimation room 1 h before testing for acclimation. During acclimation, food and water were removed and were not replaced until the end of that testing period. Testing began 2 h before their change in light cycle.

Using a clear acrylic 3 chambered association box (69 × 20 × 20 cm), with 6×5.5 cm openings in the internal dividing walls to allow access between the 3 chambers, un-scented filter papers were taped to the top of the 2 outermost chambers out of the mouse’ reach. Enough bedding was added to cover the bottom of the chamber. Mice were given 2 min of acclimation in the unscented chamber, then placed in their acclimation cages for 6 min inside the testing room. Video recordings of acclimation were later scored for zone crossings as a measure for activity level. The filter papers were taken outside of the testing room to a smell-prep station away from the mice, without disturbing the orientation of the association chamber. 50uL of rose and lemon scents were added to the designated filter papers, and then placed back in the association chamber when the mouse’s resting period was completed. The mice were then placed in the center of the association chamber and a video recording was taken of their 2 min preference test. Between mice, the association chamber was cleaned with 40% ethanol to ensure no remaining scent lingered for the next mouse, and the chamber was rotated 180 degrees to limit any side preference the mice may have had. Videos were then scored by a person blinded to treatment conditions to determine the time spent in each smell chamber during the 2 min test.

### 3.4 Statistical analysis

All graphs were made in GraphPad Prism v10.3.1. T-tests were conducted using GraphPad Prism v10.3.1 and all ANOVA analyses were conducted using SPSS29. Significance was determined using *p* < 0.05.

## 4 Results

### 4.1 Sweetener preference assessment in C57BL/6 J mice

three sweetener solutions were tested to determine which generated a passive preference in the C57BL/6 J mice (Figure 1A). Mice were distributed by sex and age across groups so that each group had 5 males and 5 females (Figure 1B). Results of univariate 2 way ANOVA indicate no effect of sex [*F*(1,24) = 2.560, *p* > 0.05] or group assignment [*F*(2,24) = 0.046, *p* > 0.05] on mouse age, showing age distributions are even across groups. Figure 1C shows the total consumption of each solution over 48 h. Results of repeated measures 2 way ANOVA indicate an interaction between group assignment and bottle content [F(2,24) = 12.228, *p* < 0.05], but no interaction between bottle content and sex [F(1,24) = 0.965, *p* > 0.05] or between bottle content, group assignment, and sex [F(2,24) = 1.753, *p* > 0.05] on consumption. Assessment of between subject effects indicate a significant effect of group assignment on consumption [F(1,24) = 11.093, *p* < 0.05], but no effect of sex [F(1,24) = 1.654, *p* > 0.05] or interactive effects of sex and group assignment [F(2,24) = 2.146, *p* > 0.05]. For the pairwise comparisons, sexes were combined. Pairwise comparisons show that both sucrose- and saccharin-assigned mice consumed more sweetened solution than water (*p* < 0.05), while sucralose mice did not (*p* > 0.05). The mice exhibited preference for sucrose and saccharin solutions over water but no preference for sucralose. Preference scores for sucrose and saccharin over water were calculated by converting total sweetened water consumption to percent of total consumption (a preference score of above 50% is considered an expression of preference). Two tailed t-test assessment shows that the sucrose-assigned mice exhibited a higher preference score than the saccharin-assigned mice (*p* < 0.05). The mice that received sucrose solution exhibited a higher preference score than the mice that received saccharin (Figure 1D). It is important to note that two of the 10 mice in the saccharin group had a preference below 50%. These results, in addition to sucrose being well established as being preferred by mice, led to sucrose being selected as the reward for the olfaction-based learned preference assessments presented here (Verharen et al., 2023; Markov, 2022; Liu et al., 2018).

### 4.2 Establishment of olfaction-based learned preference assessment protocol

Mice were trained to associate either a lemon or rose scent with a sucrose reward (Figure 2A). Three males and three females were assigned either lemon or rose as their sucrose reward (S+) scent (Figure 2B). Sex and age could not be analyzed, as the males are older than the females. Results of paired t-test analyses show that, on test day, mice spent more time in the chamber containing the S+ scent than in the chamber containing the S- scent (Figure 2C). Preference scores for the S+ scent over the S- were calculated by converting total time spent in the S+ chamber to a percentage of total time spent in scent-containing chambers, and all mice showed a preference score above 50% (Figure 2D).

### 4.3 Assessment of LPS-induced learning deficit using olfaction-based learned preference protocol

Mice were IP injected with 500ug/kg doses of either PBS or LPS 5 h before training on the first two training days (Figure 3A). Mice were assigned either lemon or rose as their S+ scent, trained for 3 days, and assessed for learned preference on day 4 (Figures 3A,B). Mice were distributed by age to PBS and LPS treatment groups for an *n* = 3 m and 2f per group (Figure 3C). Sex effects were not assessed as there were only two females in each group. Figure 3D shows the change in body weight that mice exhibited post-injections. Results of repeated measures ANOVA show a significant interaction between treatment group and percent change in body weight *F*(1.332,10.656) = 47.683 (*p* < 0.05). Pairwise comparisons show that compared to baseline, PBS treated mice have no significant change in body weight post IP injection and LPS-treated mice have a significant change in body weight at all timepoints post IP injection. LPS treated mice also have a significant change in body weight compared to PBS treated mice at all timepoints post injection. LPS treated mice lost between 15 and 19% of their original bodyweight by day 3 of the study and began recovering their bodyweight on day 4 (Figure 3D). Figure 3E shows the time spent in S+ and S- chambers on test day. Results of repeated measures ANOVA shows a significant interaction between treatment group and time spent in each chamber *F*(1,8) = 5.365 (*p* < 0.05). Pairwise comparisons show that PBS treated mice spent significantly more time in the S+ chamber than in the S- chamber (*p* < 0.05), while LPS treated mice showed no difference in time spent between chambers (*p* > 0.05). Preference scores for the S+ scent over the S- were calculated, and all five mice treated with PBS showed a preference score above 50% (Figure 3F). Results of paired t-test analyses show that LPS treated mice had lower preference scores than PBS treated mice, and only three mice scored above 50% (Figure 3F).

## 5 Discussion

The primary aim of this study was to develop and validate a learning assessment protocol that functions independently of visual cues, motor abilities, or motivational weight loss. Existing olfaction-based learning and memory protocols offered a foundation, specifically in terms of scent formulation, training schedules, and reward choices; however, modifications were essential to ensure consistent mouse participation in training sessions (Schellinck et al., 2001).

Initially, our trials confirmed that sucrose was the optimal reward for C57BL/6 J mice, outperforming artificial sweeteners like sucralose and saccharin, as demonstrated by their higher passive preference in the sweetener preference test. This is in line with the current understanding of sucrose preference in mice (Verharen et al., 2023; Markov, 2022; Liu et al., 2018). Attempts to utilize sucrose solutions as training rewards were unsuccessful, as the mice did not engage with water bottle areas or investigate cage-placed containers. However, placing sucrose paste directly on the cage floor beneath the scent paper successfully encouraged interaction. Mice would step into the sucrose paste while investigating the scent, subsequently grooming and consuming the sucrose on their paws, leading to improved training participation. Training trials were limited to 5 min to prevent overtraining behaviors, which included resting, remaining stationary, or engaging in excessive grooming without exploration.

On training day 1 of the protocol, mice began showing these signs during their third training trial. Limiting training trials to two trials of each smell (four trials total) a day resolved the issue. On test day, mice were kept in a separate room from the testing area and the smell prep station. This ensured that only the mouse being assessed for preference was being exposed to the smells at any given time. During acclimation, the mice were placed in the center chamber of the 3 chambered association chamber and monitored to ensure they discovered all 3 chambers. Due to the box being made out of clear acrylic, the mice often bumped their noses along the edge to find the opening between chambers. Video recordings of the 2-min exposure to the smells in a 3 chambered box allowed for blinded and accurate measurements of the time spent in each chamber. Using the sucrose paste to induce a passive smell preference worked, as the mice spent more time in the chamber containing the smell paired with sucrose. Thus, we achieved the first objective of this study, and established a learning assessment protocol that did not rely on visual cues, motor function, or motivational weight loss.

To validate this protocol, we used it to assess an LPS-induced learning deficit. Using previously published dosing strategies, we gave the mice an IP injection of LPS at a dose of 500ug/kg on training days 1 and 2 (Jung et al., 2023). Mice lost weight similar to previously published LPS studies, and began recovering their body weight on day 4 (Jung et al., 2023). Due to the weight loss effects of LPS administration, we limited LPS administration to only day 1 and day 2 of training, with no LPS administration on day 3 as described in section 5.3. During training, mice were monitored using the same criteria for over-training and participation as discussed earlier. LPS treated mice were observationally less energetic, but still active during training and explored the sucrose rewards when present. However, we were unable to quantify whether the treat was consumed due to the nature of the sucrose paste. On test day, PBS treated mice showed a preference for the S+ smell while LPS mice did not, and LPS mice had a lower preference score than PBS mice. In spite of weight loss being known to increase reward-seeking behavior, LPS-treated mice failed to learn the reward-based preference. Therefore, the LPS treated mice exhibited a deficit in learning capability compared to PBS treated mice. Thus, we achieved the second objective of this study and validated the utility of this olfaction-based learned preference assessment protocol to assess learning deficits due to LPS administration.

This study is limited by the number of animals assessed, as we could not assess for sex effects with the number of mice used. We also could not assess for the effects of age, as the males were older than the females, leading to the inability to separate age effects from sex differences. However, we believe that the ability of this protocol to show significant learning capabilities with low animal numbers and combined sexes to also be a potential strength. Future investigation into the limits of this protocol should assess sex and age differences using higher animal numbers to do so. Further limitations of this protocol are similar to the limitations seen in other learning assessment protocols. It is important to keep the same investigator throughout training and limit the use of personal fragrances during these assessments, as changes in personnel and scent can distract the mice from their tasks. It is also important to keep the training rooms and testing rooms free of any loud noises, changing smells, or visual disturbances as these can distract the mice and the investigator. There is also a limitation for how many mice can be run at a time. Due to the need for 3 rooms on test day (animal waiting room, testing, and scent preparation), 2 investigators at minimum (one to clean and prepare the chambers and smells, and one to run the tests), and the need to have the mice tested within the same 2-h window to avoid circadian rhythm confounds, only 9–10 mice should be run at a time. This can be resolved with multiple testing rooms and additional personnel but is a large resource requirement in that regard.

Compared to current olfactory protocols, namely fear based and olfactory preference tests that require motivational weight loss, this protocol is less stressful on both the investigators and the mouse subjects (Brown and Wong, 2007; Schellinck et al., 2001; Jones et al., 2005). This protocol also does not rely on visual cues or motor performance, unlike MWM and Rotarod tests, and avoids the inherent stress of MWM and Rotarod tests alleviating potential confounding variables in future studies (Sankowski et al., 2019; Hung and Hsueh, 2021). This protocol avoids the visual cues needed in protocols assessing olfaction based spatiotemporal memory (Aqrabawi and Kim, 2018). This protocol could be applied to other rodent models as well, and can be used in other models of learning deficits. However, this protocol is reliant on mice not having anhedonia, so it is necessary to assess preference ability in any model being assessed using this protocol. This protocol could be combined with metabolism centered treatments and assessments, as well as other learning assessments to enhance the understanding of various disease processes. For example, the mechanisms of olfaction signaling and learning are of great interest in the context of inflammation, neurodegeneration, and even in the development of cell lines derived from the olfactory neurosphere (Orecchioni et al., 2022; Irfan et al., 2024; Bhatia-Dey and Heinbockel, 2021). This protocol could allow researchers to study the effects of these mechanisms learning and memory phenotypes without needing to use fear or motivational weight loss.

In conclusion, we have established an olfactory-based learned preference protocol is a viable tool for assessing learning abilities in preclinical models that overcomes the limitations of current olfactory-based learned preference protocols by not relying on fear or weight loss as motivation. This protocol is valuable for the field of behavioral neuroscience, as it allows for assessments of learning abilities in preclinical models with auditory or visual deficits, motor impairments, or an inability to tolerate motivational weight loss.

## Data Availability

The raw data supporting the conclusions of this article will be made available by the authors, without undue reservation.
